# Supplementary material for: Development of the advised protocol for OCT study terminology and elements anterior segment OCT extension reporting guidelines (APOSTEL-AS): Study protocol
Source: PLoS One. 2025 Nov 19;20(11):e0331272. doi: 10.1371/journal.pone.0331272 (PMC12629414; doi:10.1371/journal.pone.0331272)
Supplement: S1 File — (DOCX) [file pone.0331272.s001.docx]

Supp 1: Literature search strategy

The following databases will be searched:

Medline

Embase

CINAHL

From inception

The search terms used will comprise:

| #1 | **anterior eye segments**  "anterior eye segment"[MeSH Terms] OR ("anterior"[All Fields] AND "eye"[All Fields] AND "segment"[All Fields]) OR "anterior eye segment"[All Fields] |
| --- | --- |
| #2 | **optical coherence tomography**  "tomography, optical coherence"[MeSH Terms] OR ("tomography"[All Fields] AND "optical"[All Fields] AND "coherence"[All Fields]) OR "optical coherence tomography"[All Fields] OR ("optical"[All Fields] AND "coherence"[All Fields] AND "tomography"[All Fields]) |
| #3 | #1 AND #2 |
| #4 | **anterior segment optical coherence**  ("anterior"[All Fields] OR "anteriores"[All Fields] OR "anteriorization"[All Fields] OR "anteriorized"[All Fields] OR "anteriors"[All Fields]) AND ("segment"[All Fields] OR "segment s"[All Fields] OR "segmental"[All Fields] OR "segmentally"[All Fields] OR "segmentals"[All Fields] OR "segmentation"[All Fields] OR "segmentational"[All Fields] OR "segmentations"[All Fields] OR "segmented"[All Fields] OR "segmenter"[All Fields] OR "segmenters"[All Fields] OR "segmenting"[All Fields] OR "segments"[All Fields]) AND ("eye"[MeSH Terms] OR "eye"[All Fields] OR "optic"[All Fields] OR "optic s"[All Fields] OR "optical"[All Fields] OR "optically"[All Fields] OR "optics"[All Fields]) AND ("coherence"[All Fields] OR "coherences"[All Fields] OR "coherencies"[All Fields] OR "coherency"[All Fields] OR "coherent"[All Fields] OR "coherently"[All Fields]) |
| #5 | **anterior segment oct**  ("anterior"[All Fields] OR "anteriores"[All Fields] OR "anteriorization"[All Fields] OR "anteriorized"[All Fields] OR "anteriors"[All Fields]) AND ("segment"[All Fields] OR "segment s"[All Fields] OR "segmental"[All Fields] OR "segmentally"[All Fields] OR "segmentals"[All Fields] OR "segmentation"[All Fields] OR "segmentational"[All Fields] OR "segmentations"[All Fields] OR "segmented"[All Fields] OR "segmenter"[All Fields] OR "segmenters"[All Fields] OR "segmenting"[All Fields] OR "segments"[All Fields]) AND "oct"[All Fields] |
| #6 | #3 OR #4 OR #5 |
