# Supplementary material for: Development of the advised protocol for OCT study terminology and elements anterior segment OCT extension reporting guidelines (APOSTEL-AS): Study protocol
Source: PLoS One. 2025 Nov 19;20(11):e0331272. doi: 10.1371/journal.pone.0331272 (PMC12629414; doi:10.1371/journal.pone.0331272)
Supplement: Attachment — Submitted filename: PONE-D-24-47643 APOSTEL-AS.pdf [file pone.0331272.s002.pdf]

PLOS ONE

Development of the Advised Protocol for OCT Study Terminology and Elements Anterior Segment OCT extension reporting guidelines (APOSTEL-AS): Study protocol

--Manuscript Draft--

Manuscript Number:	PONE-D-24-47643
Article Type:	Study Protocol
Full Title:	Development of the Advised Protocol for OCT Study Terminology and Elements Anterior Segment OCT extension reporting guidelines (APOSTEL-AS): Study protocol
Short Title:	APOSTEL-As Protocol
Corresponding Author:	Ameenat Lola Solebo<br>UCL GOS ICH: University College London Great Ormond Street Institute of Child Health<br>London, UNITED KINGDOM OF GREAT BRITAIN AND NORTHERN IRELAND
Keywords:	Imaging; Ocular anatomy; Reporting standards; reproducibility; Optical coherence tomography
Abstract:	Background<br><br>Anterior segment optical coherence tomography (AS-OCT) is emerging as a valuable diagnostic, monitoring and predictive tool. Clinical utility has been suggested for ophthalmic disorders such as glaucoma, corneal disease, cataract and uveitis, which taken together comprise the majority of the blinding conditions affecting working age individuals globally. The current absence of guidelines for reporting OCT studies that quantitatively assess the anterior segment is an obstacle to reproducibility and interoperability. To provide this guidance, we aim to extend the existing Advised Protocol for OCT Study Terminology and Elements (APOSTEL) guidelines, to ensure applicability to AS-OCT.<br><br>Methods<br><br>In line with EQUATOR Network guidance for the development of reporting guidelines, APOSTEL-AS will be developed through a staged consensus process involving literature review and Delphi consensus across an international multi-disciplinary stakeholder group, overseen by a multi-disciplinary multi-national Steering Committee. The systematic scoping review will be used to generate candidate items, support the development of a consensus nomenclature for AS-OCT representation of ocular structure, and to form Delphi group membership. Delphi methodology, used to consider items for inclusion, rewording or exclusion, will be undertaken in line with ACCORD (ACcurate COnsensus Reporting Document) guidance, with at least two rounds of Delphi survey, inclusion consensus threshold set at 80%, and steering committee reviews between rounds. The resultant APOSTEL-AS guideline will undergo piloting before dissemination of the final version.<br><br>Discussion<br><br>The APOSTEL-AS checklist, with minimum and recommended items to be reported about study methods, should provide timely support for researchers to ensure future standardisation, interoperability and reproducibility of reported work, hastening implementation and the translation of knowledge into clinically beneficial action.
Order of Authors:	Ameenat Lola Solebo
	Marcus Ang, PhD
	Alice Bellchambers, MBBS
	Colin J Chu, PhD
	Alastair K Denniston, FRCOphth
	Laura E Downie, PhD

Powered by Editorial Manager® and ProduXion Manager® from Aries Systems Corporation

	Thomas Evans, BSc
	Alexander S Fraser, MBBS
	Scott A. Hau, MSCOptom
	Alex S Huang, MD
	Pearse A. Keane, FRCOphth
	Xiaoxuan Liu, PhD
	Jodhbir S Mehta, PhD
	Giovanni Ometto, PhD
	Axel Petzold, MD, PhD
	Edmund Tsui, MD
	Tamara S Fraser, MBBS
	Benjamin Xu, PhD
**Additional Information:**	
**Question**	**Response**
**Financial Disclosure**<br><br>Enter a financial disclosure statement that describes the sources of funding for the work included in this submission. Review the submission guidelines for detailed requirements. View published research articles from *PLOS ONE* for specific examples.<br><br>This statement is required for submission and **will appear in the published article** if the submission is accepted. Please make sure it is accurate.<br><br>**Funded studies**<br>Enter a statement with the following details:<br>• Initials of the authors who received each award<br>• Grant numbers awarded to each author<br>• The full name of each funder<br>• URL of each funder website<br>• Did the sponsors or funders play any role in the study design, data collection and analysis, decision to publish, or preparation of the manuscript?<br><br>Did you receive funding for this work?	Yes
Please add funding details.<br>as follow-up to "**Financial Disclosure**	AL Solebo is supported by an NIHR Clinician Scientist grant (https://www.nihr.ac.uk CS-2018-18-ST2-005). All research at UCL Great Ormond Street Institute of Child

Enter a financial disclosure statement that describes the sources of funding for the work included in this submission. Review the submission guidelines for detailed requirements. View published research articles from *PLOS ONE* for specific examples.<br><br>This statement is required for submission and **will appear in the published article** if the submission is accepted. Please make sure it is accurate.<br><br>**Funded studies**<br>Enter a statement with the following details:<br>• Initials of the authors who received each award<br>• Grant numbers awarded to each author<br>• The full name of each funder<br>• URL of each funder website<br>• Did the sponsors or funders play any role in the study design, data collection and analysis, decision to publish, or preparation of the manuscript?<br><br>Did you receive funding for this work?"	Health is made possible by the NIHR Great Ormond Street Hospital Biomedical Research Centre.<br>Prof Keane is supported by a UK Research & Innovation Future Leaders Fellowship (https://www.ukri.org/what-we-do/developing-people-and-skills/future-leaders-fellowships/, MR/T019050/1) and The Rubin Foundation Charitable Trust (https://findthatcharity.uk/orgid/GB-CHC-327062)<br>The funders had no role in study design, data collection and analysis, decision to publish, or preparation of the manuscript.
Please select the country of your main research funder (please select carefully as in some cases this is used in fee calculation).<br>as follow-up to "**Financial Disclosure**<br><br>Enter a financial disclosure statement that describes the sources of funding for the work included in this submission. Review the submission guidelines for detailed requirements. View published research articles from *PLOS ONE* for specific examples.<br><br>This statement is required for submission and **will appear in the published article** if the submission is accepted. Please make sure it is accurate.	UNITED KINGDOM - GB

Powered by Editorial Manager® and ProduXion Manager® from Aries Systems Corporation

**Funded studies**<br>Enter a statement with the following details:<br>• Initials of the authors who received each award<br>• Grant numbers awarded to each author<br>• The full name of each funder<br>• URL of each funder website<br>• Did the sponsors or funders play any role in the study design, data collection and analysis, decision to publish, or preparation of the manuscript?<br><br>Did you receive funding for this work?"	
**Competing Interests**<br><br>Use the instructions below to enter a competing interest statement for this submission. On behalf of all authors, disclose any competing interests that could be perceived to bias this work—acknowledging all financial support and any other relevant financial or non-financial competing interests.<br><br>This statement is required for submission and **will appear in the published article** if the submission is accepted. Please make sure it is accurate and that any funding sources listed in your Funding Information later in the submission form are also declared in your Financial Disclosure statement.<br><br>View published research articles from *PLOS ONE* for specific examples.	I have read the journal's policy and the authors of this manuscript have the following competing interests: Dr Keane has acted as a consultant for Retina Consultants of America, Topcon, Roche, Boehringer-Ingleheim, and Bitfount and is an equity owner in Big Picture Medical. He has received speaker fees from Zeiss, Novartis, Gyroscope, Boehringer-Ingleheim, Apellis, Roche, Abbvie, Topcon, and Hakim Group. He has received travel support from Bayer, Topcon, and Roche. He has attended advisory boards for Topcon, Bayer, Boehringer-Ingleheim, RetinAI, and Novartis. No other author has competing interests.

Powered by Editorial Manager® and ProduXion Manager® from Aries Systems Corporation

**NO authors have competing interests**<br><br>Enter: *The authors have declared that no competing interests exist.*<br><br>**Authors with competing interests**<br><br>Enter competing interest details beginning with this statement:<br><br>*I have read the journal's policy and the authors of this manuscript have the following competing interests: [insert competing interests here]*<br><br>* typeset	
**Ethics Statement**<br><br>Enter an ethics statement for this submission. This statement is required if the study involved:<br><br>• Human participants<br>• Human specimens or tissue<br>• Vertebrate animals or cephalopods<br>• Vertebrate embryos or tissues<br>• Field research<br><br>Write "N/A" if the submission does not require an ethics statement.<br><br>General guidance is provided below. Consult the submission guidelines for detailed instructions. **Make sure that all information entered here is included in the Methods section of the manuscript.**	Ethics approval for this work has been granted by the University College London Institutional Review Board (UCL Research Ethics Committee Approval ID Number: 26253/001).<br>Consent: N/a

**Format for specific study types**<br><br>**Human Subject Research (involving human participants and/or tissue)**<br>• Give the name of the institutional review board or ethics committee that approved the study<br>• Include the approval number and/or a statement indicating approval of this research<br>• Indicate the form of consent obtained (written/oral) or the reason that consent was not obtained (e.g. the data were analyzed anonymously)<br><br>**Animal Research (involving vertebrate animals, embryos or tissues)**<br>• Provide the name of the Institutional Animal Care and Use Committee (IACUC) or other relevant ethics board that reviewed the study protocol, and indicate whether they approved this research or granted a formal waiver of ethical approval<br>• Include an approval number if one was obtained<br>• If the study involved *non-human primates*, add *additional details* about animal welfare and steps taken to ameliorate suffering<br>• If anesthesia, euthanasia, or any kind of animal sacrifice is part of the study, include briefly which substances and/or methods were applied<br><br>**Field Research**<br><br>Include the following details if this study involves the collection of plant, animal, or other materials from a natural setting:<br>• Field permit number<br>• Name of the institution or relevant body that granted permission	
**Data Availability**<br><br>Authors are required to make the data underlying their research findings fully available, without restriction.<br><br>If the manuscript reports pilot or preliminary results, the data underlying those results must be made available at the time of publication. A Data Availability	N/A - No results are reported

Statement describing where the data can be found is required at submission. Your answers to this question constitute the Data Availability Statement and will be published in the article, if accepted. When legal or ethical requirements prohibit public data sharing, authors must indicate how researchers may obtain access to the data. See the PLOS Data Policy and FAQ for detailed information.<br><br>**Important:** Stating 'data available on request from the author' is not sufficient. If your data are only available upon request, select 'No' for the first question and explain your situation in the text box.<br><br>For protocols without pilot or preliminary data, authors are strongly encouraged to state how they plan to share research data from their study when it is completed or published.<br><br>If your study includes pilot data, will they be made fully available without restriction?	
**Describe where the data may be found in full sentences. If you are copying our sample text, replace any instances of XXX with the appropriate details.**<br><br>• If **no pilot data are reported** in the manuscript, please enter the following: *No datasets were generated or analysed during the current study. All relevant data from this study will be made available upon study completion.*<br>• If the manuscript reports pilot data **held in a public repository**, include URLs, accession numbers or DOIs. If this information will only be available after acceptance, indicate this by ticking the box below. For example: *All XXX files are available from the XXX database (accession number(s) XXX, XXX.).*<br>• If the manuscript reports pilot data contained **within the manuscript and/or**	No datasets were generated or analysed during the current study. All relevant data from this study will be made available upon study completion.

**Supporting Information files**, enter the following:<br>*All relevant data are within the manuscript and its Supporting Information files.*<br>• If neither of these applies but you are able to provide **details of access elsewhere**, with or without limitations, please do so. For example:<br><br>*Data cannot be shared publicly because of [XXX]. Data are available from the XXX Institutional Data Access / Ethics Committee (contact via XXX) for researchers who meet the criteria for access to confidential data.*<br><br>*The data underlying the results presented in the study are available from (include the name of the third party and contact information or URL).*<br>• This text is appropriate if the data are owned by a third party and authors do not have permission to share the data.<br><br>For study protocols with data management or sharing plans, authors are encouraged to briefly describe in their Data Availability Statement how data they generate will be made accessible when the study is completed. For example:<br>• Deidentified research data will be made publicly available when the study is completed and published.<br><br>* typeset	
Additional data availability information:	Tick here if your circumstances are not covered by the questions above and you need the journal's help to make your data available.

Manuscript

Click here to access/download;Manuscript;PLOSone_R_tracked.docx

Full title:

Development of the Advised Protocol for OCT Study Terminology and Elements Anterior Segment OCT extension reporting guidelines (APOSTEL-AS): Study protocol

Short title

APOSTEL-AS extension

Authors: Ameenat L Solebo PhD[1,2], Marcus Ang PhD[3,4], Alice Bellchambers MBBS[1,5], Colin J Chu PhD[6,7], Prof Alastair K Denniston FRCOphth[8,9], Prof Laura E Downie PhD[10],Thomas Evans[11], Alexander S Fraser MD[12], Scott Hau MCOptom[6], Prof Alex S Huang MD[13], Prof Pearse A. Keane FRCOphth[6,7], Xiaoxuan Liu PhD[8,9,14], Prof Jodhbir S Mehta PhD[3,15], Giovanni Ometto PhD[5,6,16], Prof Axel Petzold MD PhD[6,17,18], Edmund Tsui MD[19], Tamara S Fraser MD[20], Prof Benjamin Xu PhD[21]

1. Population, Policy and Practice Department, UCL GOS Institute of Child Health, London, UK; Great Ormond Street Hospital, London, UK,
2. Great Ormond Street NIHR Biomedical Research Centre, London, UK
3. Cornea and External Eye Disease Service and Refractive Service, Singapore National Eye Center, Singapore
4. DUKE-NUS Department of Ophthalmology and Visual Sciences, Singapore
5. University Hospitals Sussex NHS Foundation Trust, UK
6. Moorfields NIHR Biomedical Research Centre, London, UK
7. UCL Institute of Ophthalmology, London, UK
8. NIHR Birmingham Biomedical Research Centre, Birmingham, UK
9. College of Medicine and Health, University of Birmingham, Birmingham, UK

10. Department of Optometry and Vision Sciences, The University of Melbourne, Parkville, Victoria, Australia
11. Department of Ophthalmology, Sheffield Teaching Hospitals NHS Foundation Trust, Sheffield, UK
12. Trinidad Eye Hospital, San Fernando, Trinidad and Tobago
13. Hamilton Glaucoma Center, The Viterbi Family Department of Ophthalmology, Shiley Eye Institute, University of California, San Diego, CA, USA
14. Birmingham Health Partners Centre for Regulatory Science and Innovation
15. Singapore Eye Research Institute, Singapore.
16. City St George's, University of London, Department of Optometry & Visual Sciences, London, UK.
17. University College London, UCL Institute of Neurology, London UK
18. Departments of Ophthalmology and Neurology, Amsterdam UMC, NL.
19. Ocular Inflammatory Disease Center, UCLA Stein Eye Institute, David Geffen School of Medicine at UCLA, Los Angeles, California, USA
20. Mid South Essex NHS Foundation Trust, Essex, UK
21. USC Roski Eye Institute, Keck School of Medicine at USC, Los Angeles, California, USA

Corresponding author: Ameenat Lola Solebo, UCL GOS Institute of Child Health, 30 Guilford Street, London, WC1N 1EH, UK. Email a.solebo@ucl.ac.uk

Abstract

Background:

Anterior segment optical coherence tomography (AS-OCT) is emerging as a valuable diagnostic, monitoring and predictive tool. Clinical utility has been suggested for ophthalmic disorders such as glaucoma, corneal disease, cataract and uveitis, which taken together comprise the majority of the blinding conditions affecting working age individuals globally. The current absence of guidelines for reporting OCT studies that quantitatively assess the anterior segment is an obstacle to reproducibility and interoperability. To provide this guidance, we aim to extend the existing Advised Protocol for OCT Study Terminology and Elements (APOSTEL) guidelines, to ensure applicability to AS-OCT.

Methods

In line with EQUATOR Network guidance for the development of reporting guidelines, APOSTEL-AS will be developed through a staged consensus process involving literature review and Delphi consensus across an international multi-disciplinary stakeholder group, overseen by a multi-disciplinary multi-national Steering Committee. The systematic scoping review will be used to generate candidate items, support the development of a consensus nomenclature for AS-OCT representation of ocular structure, and to form Delphi group membership. Delphi methodology, used to consider items for inclusion, rewording or exclusion, will be undertaken in line with ACCORD (ACcurate COnsensus Reporting Document) guidance, with at least two rounds of Delphi survey, inclusion consensus threshold set at 80%, and steering committee reviews between rounds. The resultant APOSTEL-AS guideline will undergo piloting before dissemination of the final version.

Discussion

The APOSTEL-AS checklist, with minimum and recommended items to be reported about study methods, should provide timely support for researchers to ensure future standardisation,

interoperability and reproducibility of reported work, hastening implementation and the translation of knowledge into clinically beneficial action.

Summary

Anterior segment optical coherence tomography (AS-OCT) is emerging as a valuable diagnostic, monitoring and predictive tool. To address the current absence of guidelines for reporting OCT studies which quantitatively assess the anterior segment, we aim to extend the existing Advised Protocol for OCT Study Terminology and Elements (APOSTEL) recommendations, to enable applicability to studies involving AS-OCT. Following steering committee formation, literature review will be used to generate items additional to those within the current APOSTEL guidance, to create a consensus nomenclature and the Delphi membership. A multi-round Delphi exercise, with the inclusion consensus threshold set at 80%, will be used to generate reporting guidance items, with process overview by the steering committee. The resultant guideline will be piloted prior to dissemination, providing a timely support tool for researchers to ensure future standardisation, interoperability and reproducibility of reported work, hastening the implementation and the translation of knowledge on the utility of AS-OCT into clinical benefit.

Introduction

Optical coherence tomography (OCT) has changed the face of ophthalmic clinical practice and medical research. The research and clinical utility of ocular OCT biomarkers has been demonstrated within ophthalmology,(1–3) and beyond the eye.(4) Quantitative data from posterior segment ocular OCT, such as retinal nerve fibre layer or central macular thickness, have been used as diagnostic, predictive, prognostic and monitoring biomarkers for inflammatory, degenerative, vascular, and metabolic disorders.(4–7)

The validation and adoption of these biomarkers has been enabled by the standardisation and harmonisation of the reporting of research findings within the underpinning evidence base. The 2016 and 2021 Advised Protocol for OCT Study Terminology and Elements (APOSTEL) and APOSTEL2.0 recommendations(8,9) for reporting quantitative retinal OCT studies strengthened reproducibility and interoperability across OCT studies of the posterior segment of the eye. However, some aspects of the current APOSTEL guidance are not applicable to anterior segment OCT (AS-OCT) studies. AS-OCT is being used to derive quantitative data from images of the normal physiology of the anterior ocular structure, data which carry clinical utility for the diagnosis and management of diseases involving those structures. These diseases include tear film disorders, corneal pathology, glaucoma, cataract and anterior uveitis,(10–16) disorders which, collectively, constitute the most common causes of working aged blindness globally.(17)

In order to improve the reproducibility and interoperability of AS-OCT studies, we aim to develop a checklist of the core information that should be provided when reporting studies in which the quantitatively assessment of AS OCT structures is undertaken. This checklist will be developed using the framework of the current APOSTEL recommendations.(8)

Materials and methods

APOSTEL-AS has been registered as a reporting guideline under development on the EQUATOR (Enhancing the QUality and Transparency Of health Research) library of reporting guidelines and will be developed in accordance with the EQUATOR Network's methodological framework. A steering committee with representation of expertise in tear film, corneal, lens, glaucoma and uveitic disorders, expertise in multinational, multi-disciplinary consensus exercises, and including investigators involved in developing the APOSTEL guidelines has been formed to oversee the conduct and methodology of the study. A multi-stage Delphi consensus approach will be used (figure 1).

Figure 1. APOSTEL-AS development process

**Further Delphi rounds may be needed to reach consensus*

Literature review and candidate item generation

An initial list of candidate items for the APOSTEL-AS checklist will be generated through review of the published literature and consultation with the steering committee (SC). A database search will be performed using the terms "anterior segment optical coherence tomography" (see supplemental document, search approach) to identify eligible studies. Eligibility criteria comprise publications reporting the analysis of quantitative data derived from AS-OCT acquired from human eyes of living participants. Specific considerations for Screening, selection AS-OCT will be identified from these studies and extracted as potential reporting items using a specific data collection form *developed de novo* by the SC. and data extraction to the data collection form (DCF) will be undertaken independently by at least two investigators. Items will be reframed as candidate reporting items ahead of the consensus

process. The SC will also create a consensus nomenclature for ocular anterior segment structures visible on axial cross sectional OCT imaging. This consensus nomenclature will be developed by the APOSTEL-AS SC using the nominal group technique, with consensus reached through discussion and majority voting.

Delphi consensus group membership:

Membership of the Delphi consensus group will be informed by the findings of the systematic review. Corresponding authors of studies reporting quantitative anterior segment OCT results will be contacted by email, requesting a response within six weeks. Reminder emails will be sent three weeks later. Records will be kept of attempted email and confirmed contact. Response rates will be reported using those who complete the Delphi as the nominator, and those for whom receipt of the email can be confirmed as the denominator. Other stakeholders to be identified and invited by the SC to join the Delphi consensus group comprise eye healthcare professionals, patients, methodologists, statisticians, computer scientists, industry representatives, health informaticists, and journal editors. Experts will be allowed to support snowball recruitment (i.e., to suggest additional experts). Records will be kept of all those successfully contacted, either directly by the SC or as corresponding authors, or indirectly through snowball recruitment.

Online Questionnaire 1 (Delphi 1):

Participants will be given written information about the study and asked to state their professional role, region of origin, and level of expertise (using years active). The candidate reporting items generated by the systematic review will be presented to the Delphi consensus group members, alongside the existing APOSTEL 2.0 items. Members will be asked to vote on each item using a 3-point scale: not important, important but not critical, and important

and critical. They will also be asked about their agreement on the wording of each item and given the opportunity to submit free text comments about suggested amendments to wording of additional items, and invited to suggest additional items.

OQ2 (Expert Group virtual meeting):
We will then form a panel of experts comprising the SC and selected members of the Delphi consensus group (ensuring representation of stakeholder groups), who will convene at a virtual meeting. The aggregated results of the initial questionnaire, comments made by participants) will be reviewed by the panel. Following open discussion of the Delphi results, anonymous electronic voting will be used to include or exclude each reworded, amended, or newly suggested item. An 80% threshold will be used to demonstrate majority consensus for the inclusion of an item.

OQ3 (Delphi 2): The included items will be considered in a second Delphi round by the original Delphi consensus group. In this round, participants will be asked to approve or reject the list of APOSTEL-AS items. Items achieving consensus ≥80% will be included. Any item which does not achieve consensus but is approved by the majority (51-79% inclusive) will be reconsidered by the expert group for consideration following further amendment or refinement, and representation in a subsequent round (Delphi 3).

OQ4 (Delphi 3): Those items that reached majority consensus but did not achieve the 80% consensus threshold will be represented to the Delphi consensus group following amendment by the SC for one final Delphi round. Participants will again be asked to approve or reject the items. Again, items achieving consensus ≥80% will be included. Any item that does not achieve consensus but is approved by the majority (51-79% inclusive) will be reconsidered

within subsequent Delphi rounds (OQ5+) by the expert group for consideration for further amendment or refinement prior to inclusion in the penultimate list.

Guideline pilot

The penultimate guideline will be checked by the Operations team (ALS, SD, AB, AF, TT) to ensure its clarity. It will then be presented to at least five stakeholders for piloting. The stakeholders participating in this pilot will comprise Delphi consensus participants who were not part of the expert group, or additional external experts identified following the start of the Delphi process. The Operations team will use feedback from this pilot to refine the guideline and produce the final version of the checklist.

Study status and timelines

Study stages up to OQ2 (expert group virtual meeting) have been completed (by date November 10th 2024) with OQ3, OQ4 and piloting due to be completed in February 2025, April 2025 and June 2025 respectively, with the result (the final tool) due in August 2025.

Ethics approval for this work has been granted by the University College London Institutional Review Board (UCL Research Ethics Committee Approval ID Number: 26253/001). Informed electronic consent will be obtained from consensus group members.

Discussion

The APOSTEL-AS recommendations will be an extension to the current APOSTEL guidelines, adding specific parameters for the reporting of AS-OCT data. AS-OCT is increasingly being used to derive quantitative data from images of normal physiology and for

diseases involving the anterior segment of the eye. There are several differences in the acquisition and analysis of anterior versus posterior segment OCT images. Whilst posterior segment OCT (PS-OCT) concerns images of tissues with similar refractive indices and typically measuring no more than 1mm in depth (as measured from the inner limiting membrane to the sclera), with the *'en face'* area of interest typically being $4mm^2$, the ocular structures imaged in AS-OCT are arranged over a greater depth (at least 10mm, as measured from the corneal epithelium to the anterior hyaloid area) and with a much wider area of interest depending on the clinical or research need.(18) Furthermore, whilst the posterior segment tissues minimally scatter light, there are unique features related to light transmissibility of the different anterior ocular structures, from the optically clear cornea to the optically opaque sclera.(18) These considerations differentiate AS-OCT from PS-OCT, but it is also likely that these challenges result in heterogeneity of study methods, including acquisition processes, image quality assessment, data extraction and image analysis across the AS-OCT literature. APOSTEL-AS will provide timely support for users of AS-OCT, to ensure future standardisation, interoperability and reproducibility of reported research and clinical findings.

A limitation of the approach described in this protocol is the restriction of the identification of potential consensus group members to those researchers who have published eligible studies within the preceding four years. High impact, novel, innovative imaging studies may require lengthy recruitment periods, which may mean that our time-restricted approach does not involve scientists whose work is not yet disseminated through peer-reviewed publications. The use of 'snowball' extension (i.e. consensus group members identifying other potential members) should help to address this limitation. Strengths of the approach include the involvement of a wide range of stakeholder expertise and experience, across

multiple countries, with collection of information about their degree of experience and expertise to inform the contextualisation of responses. Development of items will be underpinned by a comprehensive systematic review, with collection of data from manuscripts published over the last 20 years. It is acknowledged that this comprehensiveness may result in the collection of historical data from imaging platforms that are no longer in use, due to being superseded by more contemporary devices.

Several consensus methods exist for the capture and description of consensus across multi-disciplinary groups. The nominal group technique (NGT) was selected for the initial activities of the SC, in view of its structured sequence of events, principal of equal participation, and promotion of diversity in opinion, which should support the work of this smaller group.(19) NGT consensus exercises can be time consuming, particularly for large groups, but this process will benefit from facilitation undertaken by subject matter experts with experience in supporting consensus initiatives.(20,21) The selection of the Delphi method for the development of the checklist enables this activity to be undertaken by a large, geographically and scientifically diverse group with different degrees and spheres of experience and expertise. A recently published EQUATOR (Enhancing the QUality and Transparency Of health Research) guideline for reporting consensus-based methods in biomedical research and clinical practice (the ACcurate COnsensus Reporting Document, or ACCORD) will be used to report the study findings.(19)

The original APOSTEL checklist was developed following concerns around the absence of consensus around quality control criteria for posterior segment OCT imaging.(9) Similar concerns are emerging for AS-OCT imaging, with recognition of this issue being a particular obstacle to the potentially transformative role of artificial intelligence (AI) approaches to

image analysis and the development of imaging-based biomarkers.(22,23) AI approaches hold the promise of dramatically increasing the utility of clinical images, with deep learning (DL) based image analysis in particular proving non-inferior to expert human assessment.(12,24,25) Human analysis of the quantitative information that can be extracted from anterior segment structures is subjective, open to inter- and intraobserver variability, and time consuming.(26) DL image analysis methods would provide objective, repeatable, reliable OCT biomarkers, but these approaches require large (or harmonised), diverse, annotated datasets. The current absence of explicit and comprehensive reporting of AS-OCT study methods is an obstacle to comparison or collation of existing annotated imaging datasets. Through the wide dissemination and uptake of the APOTEL-AS guidance, enabled by ensuring accessibility in open access formats and translation into other languages as directed and supported by stakeholders, this obstacle should be addressed.

Authors' contributions

Conceptualisation and manuscript drafting: AL Solebo

All authors contributed to study design, manuscript editing and final approval for submission.

Acknowledgments

We thank the Enhancing the Quality and Transparency of health research Network (EQUATOR Network) team for their support with study development.

References

1. Hui VWK, Szeto SKH, Tang F, Yang D, Chen H, Lai TYY, et al. Optical Coherence Tomography Classification Systems for Diabetic Macular Edema and Their

Associations With Visual Outcome and Treatment Responses - An Updated Review. Asia Pac J Ophthalmol (Phila). 2022 Jun 1;11(3):247–57.

2. Hood DC, La Bruna S, Tsamis E, Thakoor KA, Rai A, Leshno A, et al. Detecting glaucoma with only OCT: Implications for the clinic, research, screening, and AI development. Prog Retin Eye Res. 2022 Sep;90:101052.

3. Virgili G, Menchini F, Casazza G, Hogg R, Das RR, Wang X, et al. Optical coherence tomography (OCT) for detection of macular oedema in patients with diabetic retinopathy. Cochrane Database Syst Rev. 2015 Jan 7;1:CD008081.

4. Wagner SK, Fu DJ, Faes L, Liu X, Huemer J, Khalid H, et al. Insights into Systemic Disease through Retinal Imaging-Based Oculomics. Transl Vis Sci Technol. 2020 Feb 12;9(2):6.

5. Bostan M, Li C, Sim YC, Bujor I, Wong D, Tan B, et al. Combining retinal structural and vascular measurements improves discriminative power for multiple sclerosis patients. Ann N Y Acad Sci. 2023 Sep 1;

6. Chen S, Zhang D, Zheng H, Cao T, Xia K, Su M, et al. The association between retina thinning and hippocampal atrophy in Alzheimer’s disease and mild cognitive impairment: a meta-analysis and systematic review. Front Aging Neurosci. 2023;15:1232941.

7. Wagner SK, Romero-Bascones D, Cortina-Borja M, Williamson DJ, Struyven RR, Zhou Y, et al. Retinal Optical Coherence Tomography Features Associated With Incident and Prevalent Parkinson Disease. Neurology. 2023 Aug 21;

8. Aytulun A, Cruz-Herranz A, Aktas O, Balcer LJ, Balk L, Barboni P, et al. APOSTEL 2.0 Recommendations for Reporting Quantitative Optical Coherence Tomography Studies. Neurology. 2021 Jul 13;97(2):68–79.

9. Cruz-Herranz A, Balk LJ, Oberwahrenbrock T, Saidha S, Martinez-Lapiscina EH, Lagreze WA, et al. The APOSTEL recommendations for reporting quantitative optical coherence tomography studies. Neurology. 2016 Jun 14;86(24):2303–9.

10. Chen C, Lee Y, Gerber MJ, Cheng H, Yang Y, Govetto A, et al. Intraocular robotic interventional surgical system (IRISS): Semi-automated OCT-guided cataract removal. The International Journal of Medical Robotics and Computer Assisted Surgery. 2018 Dec 28;14(6).

11. Sharma R, Sharma A, Arora T, Sharma S, Sobti A, Jha B, et al. Application of anterior segment optical coherence tomography in glaucoma. Surv Ophthalmol. 2014 May;59(3):311–27.

12. Santos VA dos, Schmetterer L, Stegmann H, Pfister M, Messner A, Schmidinger G, et al. CorneaNet: fast segmentation of cornea OCT scans of healthy and keratoconic eyes using deep learning. Biomed Opt Express. 2019 Feb 1;10(2):622.

13. Etherton K, Rahi JS, Petrushkin H, Dick AD, Akbarali S, Pattani R, et al. Quantitative and qualitative assessment of anterior segment optical coherence tomography capture of disease state in childhood anterior uveitis. Br J Ophthalmol. 2023 Jul;107(7):966–72.

14. Tsui E, Chen JL, Jackson NJ, Leyva O, Rasheed H, Baghdasaryan E, et al. Quantification of Anterior Chamber Cells in Children With Uveitis Using Anterior Segment Optical Coherence Tomography. Am J Ophthalmol. 2022 Sep;241:254–61.

15. Bao YK, Xu BY, Friedman DS, Cho A, Foster PJ, Jiang Y, et al. Biometric Risk Factors for Angle Closure Progression After Laser Peripheral Iridotomy. JAMA Ophthalmol. 2023 Jun 1;141(6):516–24.

16. Xu BY, Friedman DS, Foster PJ, Jiang Y, Porporato N, Pardeshi AA, et al. Ocular Biometric Risk Factors for Progression of Primary Angle Closure Disease: The Zhongshan Angle Closure Prevention Trial. Ophthalmology. 2022 Mar;129(3):267–75.
17. Flaxman SR, Bourne RRA, Resnikoff S, Ackland P, Braithwaite T, Cicinelli M V, et al. Global causes of blindness and distance vision impairment 1990–2020: a systematic review and meta-analysis. Lancet Glob Health. 2017 Dec;5(12):e1221–34.
18. Xie X, Corradetti G, Song A, Pardeshi A, Sultan W, Lee JY, et al. Age- and refraction-related changes in anterior segment anatomical structures measured by swept-source anterior segment OCT. PLoS One. 2020 Oct 23;15(10):e0240110.
19. Gattrell WT, Hungin AP, Price A, Winchester CC, Tovey D, Hughes EL, et al. ACCORD guideline for reporting consensus-based methods in biomedical research and clinical practice: a study protocol. Res Integr Peer Rev. 2022 Dec 7;7(1):3.
20. Solebo AL, Rahi JS, Dick AD, Ramanan A V, Ashworth J, Edelsten C. Areas of agreement in the management of childhood non-infectious chronic anterior uveitis in the UK. British Journal of Ophthalmology. 2020 Jan;104(1):11–6.
21. Solebo AL, Kellett S, Rahi J, Pattani R, Edelsten C, Dick AD, et al. Development of a Nationally Agreed Core Clinical Dataset for Childhood Onset Uveitis. Front Pediatr. 2022 Jun 21;10.
22. Tewarie P, Balk L, Costello F, Green A, Martin R, Schippling S, et al. The OSCAR-IB Consensus Criteria for Retinal OCT Quality Assessment. PLoS One. 2012 Apr 19;7(4):e34823.
23. Petzold A, Albrecht P, Balcer L, Bekkers E, Brandt AU, Calabresi PA, et al. Artificial intelligence extension of the OSCAR-IB criteria. Ann Clin Transl Neurol. 2021 Jul 19;8(7):1528–42.

24. Bolo K, Apolo Aroca G, Pardeshi AA, Chiang M, Burkemper B, Xie X, et al. Automated expert-level scleral spur detection and quantitative biometric analysis on the ANTERION anterior segment OCT system. Br J Ophthalmol. 2024 May 21;108(5):702–9.

25. Xu BY, Chiang M, Pardeshi AA, Moghimi S, Varma R. Deep Neural Network for Scleral Spur Detection in Anterior Segment OCT Images: The Chinese American Eye Study. Transl Vis Sci Technol. 2020 Mar;9(2):18.

26. Akbarali S, Rahi JS, Dick AD, Parkash K, Etherton K, Edelsten C, et al. Imaging-Based Uveitis Surveillance in Juvenile Idiopathic Arthritis: Feasibility, Acceptability, and Diagnostic Performance. Arthritis Rheumatol. 2021 Feb;73(2):330–5.

Supporting documents

Supplemental document 1: Literature search strategy

Figure 1

Click here to access/download;Figure;ApostelFig1.tif

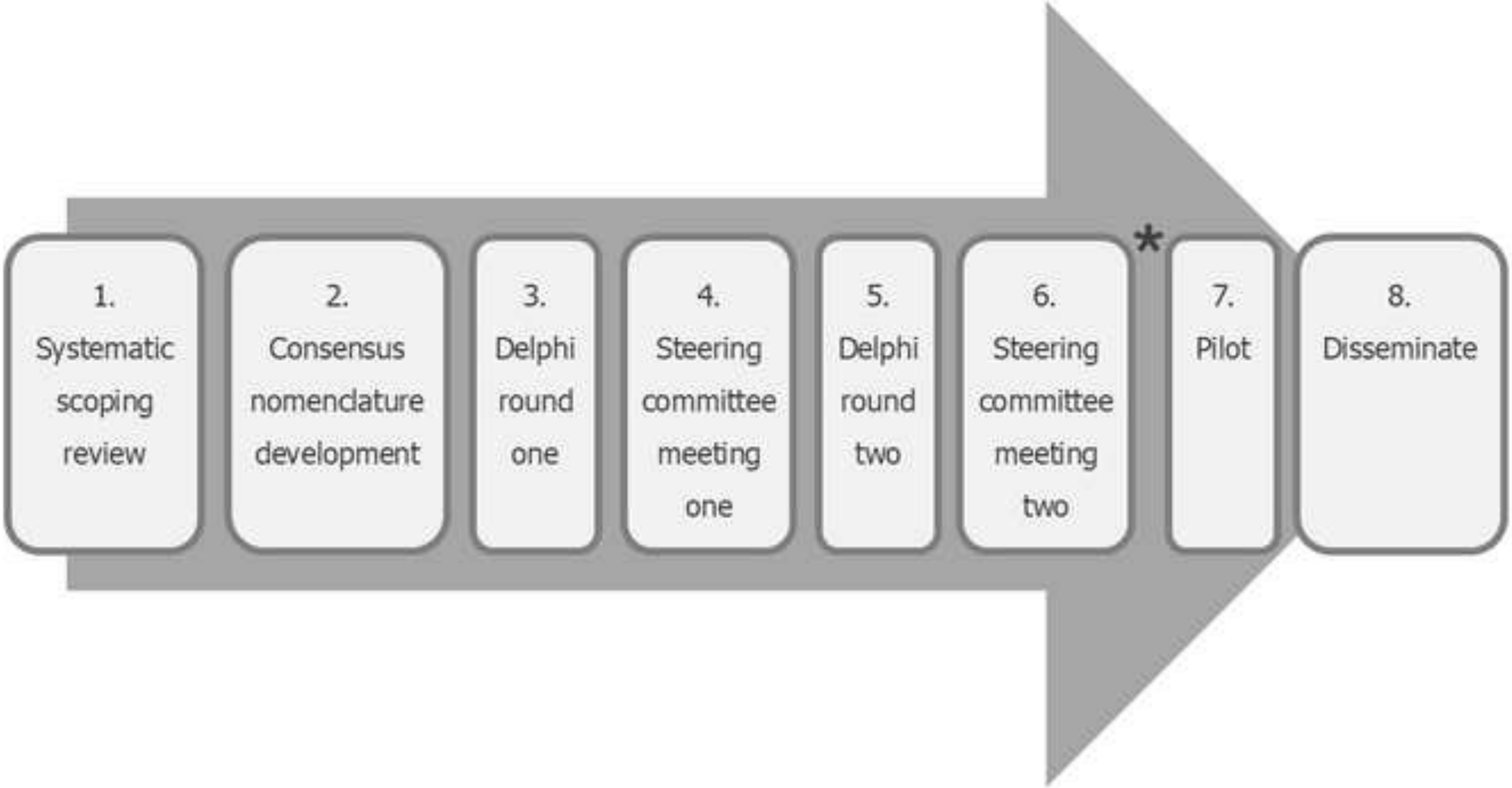

Click here to access/download
Supporting Information
Supp 1.docx
