# Supplementary material for: Development of the advised protocol for OCT study terminology and elements anterior segment OCT extension reporting guidelines (APOSTEL-AS): Study protocol
Source: PLoS One. 2025 Nov 19;20(11):e0331272. doi: 10.1371/journal.pone.0331272 (PMC12629414; doi:10.1371/journal.pone.0331272)
Supplement: Attachment — Submitted filename: PLOS_APOSTEL_REVIEWERS_PROTOCOL .docx [file pone.0331272.s004.docx]

1. Please ensure that your manuscript meets PLOS ONE's style requirements, including those for file naming. The PLOS ONE style templates can be found at

<https://journals.plos.org/plosone/s/file?id=wjVg/PLOSOne_formatting_sample_main_body.pdf> and

[https://journals.plos.org/plosone/s/file?id=ba62/PLOSOne_formatting_sample_title_authors_affiliations.pdf](https://eur01.safelinks.protection.outlook.com/?url=https%3A%2F%2Ftrack.editorialmanager.com%2FCL0%2Fhttps%3A%252F%252Fjournals.plos.org%252Fplosone%252Fs%252Ffile%253Fid%3Dba62%252FPLOSOne_formatting_sample_title_authors_affiliations.pdf%2F1%2F010f0197bfb1d600-fe93b1ba-1271-4a45-904c-a24f25fec54e-000000%2Fox3NrCbVqwoWqomuSnmpCdZVttnUMZECkg77YErHG5o%3D217&data=05%7C02%7Ca.solebo%40ucl.ac.uk%7C584dc49aa2b8433c7ad108ddb7a60b74%7C1faf88fea9984c5b93c9210a11d9a5c2%7C0%7C0%7C638868647691290239%7CUnknown%7CTWFpbGZsb3d8eyJFbXB0eU1hcGkiOnRydWUsIlYiOiIwLjAuMDAwMCIsIlAiOiJXaW4zMiIsIkFOIjoiTWFpbCIsIldUIjoyfQ%3D%3D%7C0%7C%7C%7C&sdata=2PvA7WkZzksjvIwl07%2FMrW7rab7rffo8LEG4vjbHINs%3D&reserved=0)

Author response: We have followed the provided file naming guidance for figures and supplemental data, and ensured our manuscript formatting with the PLOS ONE template provided

2. Please expand the acronym “NIHR” (as indicated in your financial disclosure) so that it states the name of your funders in full.
This information should be included in your cover letter; we will change the online submission form on your behalf.

Author response: This has been done

3. We note that the grant information you provided in the ‘Funding Information’ and ‘Financial Disclosure’ sections do not match.

When you resubmit, please ensure that you provide the correct grant numbers for the awards you received for your study in the ‘Funding Information’ section.

Author response: We have ensured that grant references are labelled as such (as one of the reference numbers in this statement relates to the funding organisation, the Rubin Foundation, rather than a specific grant)

4. Please provide a complete Data Availability Statement in the submission form, ensuring you include all necessary access information or a reason for why you are unable to make your data freely accessible. If your research concerns only data provided within your submission, please write "All data are in the manuscript and/or supporting information files" as your Data Availability Statement.

Author response: This has been done - we have stated that "All data are in the manuscript and/or supporting information files".

5. Thank you for stating the following in the Competing Interests/Financial Disclosure section:
I have read the journal's policy and the authors of this manuscript have the following competing interests: Dr Keane has acted as a consultant for Retina Consultants of America, Topcon, Roche, Boehringer-Ingleheim, and Bitfount and is an equity owner in Big Picture Medical. He has received speaker fees from Zeiss, Novartis, Gyroscope, Boehringer-Ingleheim, Apellis, Roche, Abbvie, Topcon, and Hakim Group. He has received travel support from Bayer, Topcon, and Roche. He has attended advisory boards for Topcon, Bayer, Boehringer-Ingleheim, RetinAI, and Novartis. No other author has competing interests.

We note that one or more of the authors have an affiliation to the commercial funders of this research study : Retina Consultants of America, Topcon, Roche, Boehringer-Ingleheim, Bitfount

Author response: This study has not been funded by any commercial organisations – we have now separated the funder details from the competing interests details to make this clear.

a. Please provide an amended Funding Statement declaring this commercial affiliation, as well as a statement regarding the Role of Funders in your study. If the funding organization did not play a role in the study design, data collection and analysis, decision to publish, or preparation of the manuscript and only provided financial support in the form of authors' salaries and/or research materials, please review your statements relating to the author contributions, and ensure you have specifically and accurately indicated the role(s) that these authors had in your study. You can update author roles in the Author Contributions section of the online submission form.

Please also include the following statement within your amended Funding Statement.
“The funder provided support in the form of salaries for authors [insert relevant initials], but did not have any additional role in the study design, data collection and analysis, decision to publish, or preparation of the manuscript. The specific roles of these authors are articulated in the ‘author contributions’ section.”
If your commercial affiliation did play a role in your study, please state and explain this role within your updated Funding Statement.

Author response: We have ensured that it is clear that there was no commercial funding for the study, and that the non-commercial funders had no role in the study


b. Please also provide an updated Competing Interests Statement declaring this commercial affiliation along with any other relevant declarations relating to employment, consultancy, patents, products in development, or marketed products, etc.

Within your Competing Interests Statement, please confirm that this commercial affiliation does not alter your adherence to all PLOS ONE policies on sharing data and materials by including the following statement: "This does not alter our adherence to PLOS ONE policies on sharing data and materials.” (as detailed online in our guide for authors <http://journals.plos.org/plosone/s/competing-interests>). If this adherence statement is not accurate and there are restrictions on sharing of data and/or materials, please state these. Please note that we cannot proceed with consideration of your article until this information has been declared.

Please include both an updated Funding Statement and Competing Interests Statement in your cover letter. We will change the online submission form on your behalf.

Author response: we have now separated the funder details from the competing interests details

6. Please review your reference list to ensure that it is complete and correct. If you have cited papers that have been retracted, please include the rationale for doing so in the manuscript text, or remove these references and replace them with relevant current references. Any changes to the reference list should be mentioned in the rebuttal letter that accompanies your revised manuscript. If you need to cite a retracted article, indicate the article’s retracted status in the References list and also include a citation and full reference for the retraction notice.

Author response: We confirm that the reference list is complete and correct

Reviewer #1: 

The "snowball extension" concept will be critically important to include 'persons of interest' who have expertise but may not have published in past 4 years - especially because the research output has been lower due to worldwide pandemic.
Furthermore, it is important to contact and attempt re-contact the corresponding authors as suggested in your study because things can get "lost' in the email deluge that we all are accustomed to.
Consensus opinions will have greater acceptance and adoption with inclusivity of all the expert players.

Author response: We thank the reviewer for their support for our methods (use of snowball recruitment as described in line 152, use of reminder emails as described in line 149, and the consensus approach). We note that no change has been suggested

Reviewer #2: 1.What is the motivation for this publication? This is the study protocol and one would normally want to publish the results of the study rather than the study protocol.

Author response: We thank the reviewer for asking us to explicitly state that the purpose of publishing the protocol is / was* to take an ‘Open Science’ approach to support and to support engagement for our activities across the stakeholder community of researchers and clinicians.

(*We have amended timelines within the article as the protocol was originally submitted some time ago.)

2.The methodology looks very appropriate for the main objective of the study but correct the highlighted errors as indicated in the attached copy of the manuscript.

Line 114 This should be 'quantitative'

Author response: This has been amended, thank you

Line 134 Elaborate this a little bit more. A busy reader may not have the time to refer to the supplemental document.

Author response: More details have now been provided

Line 136, “Specific considerations for Screening, selection AS-OCT”: This doesn't sound meaningful. Can you rephrase it?

Author response: Changed to “Specific considerations for acquisition, selection, processing and analyses of AS-OCT will be identified from these studies”, thank you for noting this error

Line 157 this should be 'numerator'

Author response: This has been amended, thank you
